# Bursts of CO_2_ released during freezing offer a new perspective on avoidance of winter embolism in trees

**DOI:** 10.1093/aob/mcu190

**Published:** 2014-09-24

**Authors:** A. Lintunen, L. Lindfors, P. Kolari, E. Juurola, E. Nikinmaa, T. Hölttä

**Affiliations:** 1 Department of Forest Sciences, University of Helsinki, Post Office Box 27, FI-00014, Helsinki, Finland; 2 Department of Physics, University of Helsinki, Post Office Box 64, FI-00014, Helsinki, Finland

**Keywords:** Bubble formation, cavitation, CO_2_ efflux, freezing propagation, Norway spruce, *Picea abies*, *Pinus sylvestris*, Scots pine, winter embolism, wood respiration

## Abstract

**Background and Aims:**

Woody plants can suffer from winter embolism as gas bubbles are formed in the water-conducting conduits when freezing occurs: gases are not soluble in ice, and the bubbles may expand and fill the conduits with air during thawing. A major assumption usually made in studies of winter embolism formation is that all of the gas dissolved in the xylem sap is trapped within the conduits and forms bubbles during freezing. The current study tested whether this assumption is actually valid, or whether efflux of gases from the stem during freezing reduces the occurrence of embolism.

**Methods:**

CO_2_ efflux measurements were conducted during freezing experiments for saplings of three Scots pine (*Pinus sylvestris*) and three Norway spruce (*Picea abies*) trees under laboratory conditions, and the magnitudes of the freezing-related bursts of CO_2_ released from the stems were analysed using a previously published mechanistic model of CO_2_ production, storage, diffusion and efflux from a tree stem. The freezing-related bursts of CO_2_ released from a mature Scots pine tree growing in field conditions were also measured and analysed.

**Key Results:**

Substantial freezing-related bursts of CO_2_ released from the stem were found to occur during both the laboratory experiments and under field conditions. In the laboratory, the fraction of CO_2_ released from the stem ranged between 27 and 96 % of the total CO_2_ content within the stem.

**Conclusions:**

All gases dissolved in the xylem sap are not trapped within the ice in the stem during freezing, as has previously been assumed, thus adding a new dimension to the understanding of winter embolism formation. The conduit water volume not only determines the volume of bubbles formed during freezing, but also the efficiency of gas efflux out of the conduit during the freezing process.

## INTRODUCTION

Winter embolism influences tree survival and growth in all regions where sub-zero temperatures occur. Winter embolism has been observed in numerous tree species including conifers (Sperry and Sullivan, 1992; Sparks *et al*., 2001; Mayr *et al*., 2002, 2007; Pittermann and Sperry, 2003, 2006; Mayr and Sperry, 2010) and angiosperms (Cochard and Tyree, 1990; Just and Sauter, 1991; Sperry and Sullivan, 1992; Utsumi *et al*., 1998; Nardini *et al*., 2000). Winter embolism follows from the formation of gas bubbles during freezing and their subsequent expansion during thawing (Sucoff, 1969; Ewers, 1985; Sperry and Sullivan, 1992; Davis *et al*., 1999; Mayr and Sperry, 2010). Gases dissolved in the xylem sap, including CO_2_, are not soluble in ice and are believed to be forced to form bubbles as the xylem sap freezes. Upon thawing, the bubbles released from the ice may expand and embolize the xylem conduits.

According to the LaPlace law, the fate of gas bubbles during thawing, i.e. whether they collapse or expand to embolize xylem conduits, is dependent on their size and on the pressure of the surrounding xylem sap (Pittermann and Sperry, 2006). The size of the bubbles formed during freezing is further hypothesized to correlate positively with conduit diameter (Sperry and Sullivan, 1992; Davis *et al*., 1999; Pittermann and Sperry, 2003, 2006). The link between conduit size and winter embolism has been experimentally quantified in several tree species (Sperry and Sullivan, 1992; Sperry *et al*., 1994; Davis *et al*., 1999; Pittermann and Sperry, 2003; Wheeler *et al*., 2005; Wilson and Jackson, 2006), whereas the link between conduit diameter and bubble size is only theoretical (Pittermann and Sperry, 2006) and, to our knowledge, has not been directly measured.

The basic idea behind the relationship between conduit and bubble diameter is that air is forced out of the freezing xylem sap, forming centrally located air bubbles in ice, whose volume is proportional to the cross-sectional area of a xylem conduit (Sperry and Sullivan, 1992; Pittermann and Sperry, 2006). One major assumption usually made in connection with winter embolism formation is that all of the gas dissolved in the xylem sap is trapped within the conduits and forms bubbles during freezing. In our study we tested whether this assumption is actually valid. During freezing, ice spreads rapidly inside trees (Kitaura, 1967; Hacker and Neuner, 2007; Pramsohler *et al*., 2012), which can be assumed to concentrate the dissolved gases in front of the moving ice front (Sevanto *et al*., 2012), creating a large concentration difference between the gas within the conduits and the gas in inter-conduit spaces and further in the ambient air. This increased concentration difference can be expected to accelerate the diffusion of gases out from the stem until ice has spread throughout the entire stem.

The amount of gases trapped within the xylem conduits is crucial for the size of the bubbles formed during freezing (Sperry and Sullivan, 1992; Davis *et al*., 1999; Pittermann and Sperry, 2003, 2006). The likelihood of winter embolism during thawing should decrease if gases are able to diffuse out from the conduits during the freezing process. We conducted laboratory measurements with Scots pine (*Pinus sylvestris*) and Norway spruce (*Picea abies*) seedlings to quantify the stem CO_2_ efflux during freezing, and evaluated the fraction of the freezing-related CO_2_ burst released from the xylem using a previously published mechanistic model of CO_2_ production, storage, diffusion and efflux from a tree stem (Hölttä and Kolari, 2009). Furthermore, we measured the CO_2_ efflux pattern during freezing and thawing in a mature Scots pine tree in field conditions. The prevailing understanding of winter embolism formation is improved in light of these results.

## MATERIAL AND METHODS

### Laboratory experiments

The laboratory measurements were conducted at the University of Helsinki facilities in February 2013 with three Scots pine (*Pinus sylvestris* L.) saplings and three Norway spruce (*Picea abies* L. Karst.) saplings grown in 3-L pots. The saplings were winter acclimated as they had been kept outdoors since autumn 2012. The pine saplings were 3 years old and their average base diameter was 0·80 cm. The spruce saplings were 5 years old and their average base diameter was 0·83 cm.

**Fig. 1.**
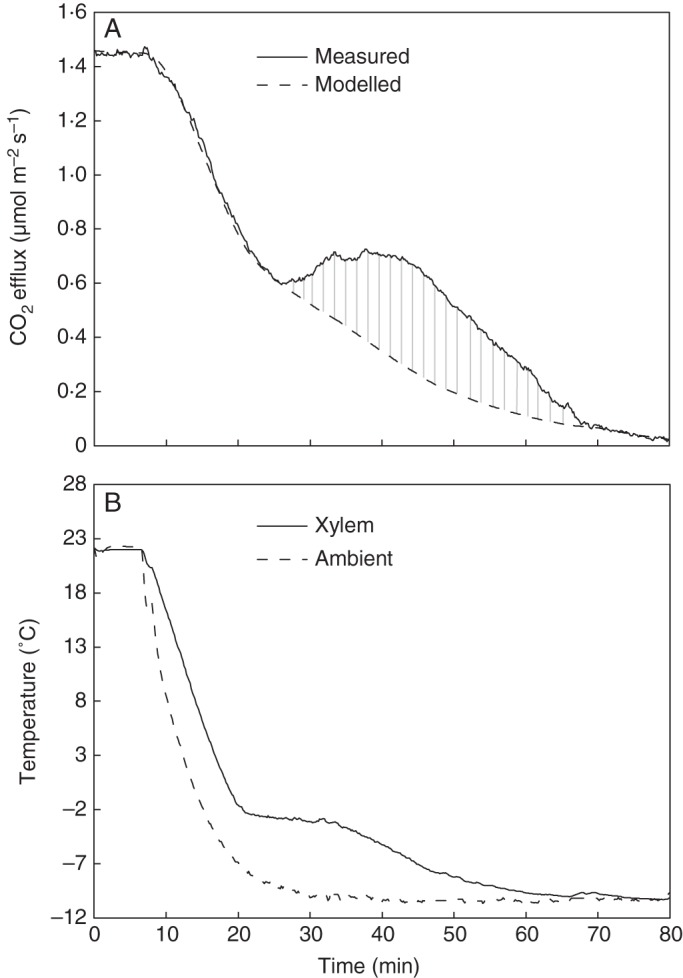
An example of a freezing experiment time series for a pine (tree no. 3 in Table 1). (A) Measured CO_2_ efflux during the freezing experiment is shown together with a modelled estimate for stem CO_2_ release if respiration was the only source of CO_2_. The integral between the measured CO_2_ efflux and modelled CO_2_ release is the freezing-related CO_2_ burst out of the stem, which is shown in grey. (B) Xylem and climate chamber temperatures measured during freezing.

The saplings were first allowed to thaw for 2 d at 6 °C. Relative air humidity was on average 77 % and the light level was approximately half of the ambient outdoor level. Half an hour before beginning the experiment, the saplings were brought to room temperature (approximately 20 °C). The experiments were conducted inside a dark climate chamber (Weiss Umwelttechnik WK11 −340/40, Vienna, Austria). Stem temperature was measured at 10-s intervals with thermocouples inserted a few millimetres inside the stem sapwood, just above the cuvette measuring CO_2_ efflux. The chamber air temperature was decreased from room temperature to –10 °C within 21–40 min. Water freezing dynamics within the xylem conduits was detected with simultaneous ambient air and stem temperature measurements. Freezing onset could be detected from an exotherm, i.e. heat release during freezing (e.g. Burke *et al*., 1976). An exotherm, and thus the timing of freezing, can be identified from our measurements as a sudden increase in the difference between xylem and ambient air temperatures due to the energy released from freezing (e.g. Fig. 1; Burke *et al*., 1976). An endotherm, i.e. the absorption of heat during thawing, can be detected from the measurements as a decrease in the difference between xylem and ambient air temperatures (Burke *et al*., 1976).

The CO_2_ efflux measurements were conducted with a portable gas exchange measuring system (GFS-3000; Heinz Walz GmbH, Effeltrich, Germany) connected to a custom-made cuvette. The opaque plastic cuvette was 8·8 cm high and 5 cm wide and consisted only of a single intact cylinder-shaped piece. The cuvette was air-tightly fixed on a stem at a height of 10 cm using rubber socks from both open ends. Due to the cuvette design, the whole seedling (apart from the pot) had to be gently forced through the cuvette (and through the rubber socks). Cuvette leakage was tested before each experimental run by checking that the two rotameters in the GFS-3000 system showed an equal value (Anon., 2012). The signal to noise ratio was generally high in the measurements, although a strong air current was detected inside the climate chamber (related to the temperature control of the chamber).

### Laboratory measurements analyses model

Stem CO_2_ efflux can be directly observed from our measurements, but the magnitude of the freezing-related CO_2_ burst cannot. This is because the CO_2_ concentration and its radial gradient within the stem are constantly changing due to CO_2_ production by respiration, radial diffusion and efflux into the ambient air. If at any moment the rate of respiration is different from the rate of efflux out of the stem, the amount of CO_2_ within the stem changes. Stem CO_2_ efflux and respiration are rather tightly coupled to each other above freezing temperatures, especially in conditions of low transpiration rates (see Teskey and McGuire, 2007; Bloemen *et al*., 2013). However, during freezing they are clearly decoupled. We used a previously published dynamic model of CO_2_ mass balance and transport within the stem presented by Hölttä and Kolari (2009) to separate the freezing-related CO_2_ burst from the total stem CO_2_ efflux and to estimate the amount of CO_2_ within the stem just prior to freezing. The difference between the measured total CO_2_ efflux and the modelled CO_2_ efflux (the model does not simulate freezing) represents the burst of CO_2_ released from the stem due to the freezing process. Accordingly, a freezing-related burst of CO_2_ from the stem occurs when the difference between measured efflux and modelled CO_2_ release is positive.

Briefly, the model (presented in detail by Hölttä and Kolari, 2009) solves the CO_2_ concentration profile within the stem by taking into account the CO_2_ production by respiration, its partitioning between the liquid and gaseous phase, and its radial diffusion according to the concentration gradient within the stem and out through the bark. The stem is radially divided into the functional components of sapwood, cambium, phloem and outer bark, each of which has its own temperature-dependent CO_2_ production rate. The stem is discretized into 25 radial elements for the numerical solution. Model parameterization was kept the same as in the original model that is parameterized for Scots pine, with the exceptions of the radial diffusion coefficient value, the absolute value of respiration, and the relative proportions of water, air and wood within the stem. Values of radial diffusion coefficient and absolute respiration were both fitted so that the dynamics and absolute values of the modelled CO_2_ efflux rate matched the measured CO_2_ efflux rate while the stem was unfrozen. The radial diffusion coefficient of CO_2_ was fitted at 75 % of the water in pine and 94 % of the water in spruce. The air and water phases were made to account for 25 and 50 % of the stem volumes, respectively (see Gartner *et al*., 2004). Henry's law coefficient, which determines the partitioning of CO_2_ between the liquid and gaseous phases, was additionally made temperature-dependent (Denbigh, 1971; Juurola *et al*., 2005). A constant pH of 5·6 was used for the Henry's law calculations (see Aubrey *et al*., 2011; Erda *et al*., 2014). The pH used in this study was similar to that reported for *Pinus sylvestris* (Perks *et al*., 2002) and *Pinus taeda* (Carter and Larsen, 1965). As the solubility of CO_2_ remains relatively constant when the pH varies from 5 to 6, a pH of pure water could be used. A Q10 value of 2·5 was used for the temperature dependency of respiration, as in the original parameterization. Sap velocity was assumed to be zero due to the low temperature and light levels during the laboratory experiment, and ambient CO_2_ concentration inside the climate chamber was measured at approx. 500 p.p.m. (0·02 mol m^−3^ at 0 °C).

**Fig. 2.**
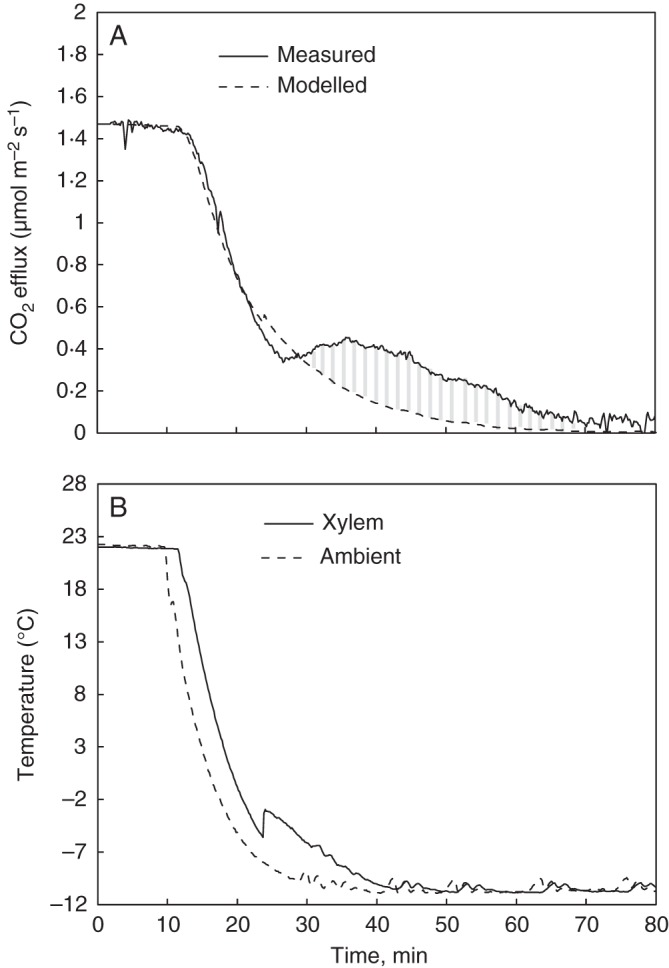
An example of a freezing experiment time series for a spruce (tree no. 6 in Table 1). (A) Measured CO_2_ efflux during the freezing experiment is shown together with a modelled estimate for stem CO_2_ release if respiration was the only source of CO_2_. The integral between the measured CO_2_ efflux and modelled CO_2_ release is the freezing-related CO_2_ burst out of the stem, which is shown in grey. (B) Xylem and climate chamber temperatures measured during freezing.

Very little information exists on the temperature dependence of the respiration rate in a frozen stem. We therefore assumed that respiration decreased linearly during freezing propagation down to the level measured after the CO_2_ efflux had settled to a constant level in a frozen stem (Figs 1A and 2A). Results with alternative respiration approaches in a frozen stem are presented in the Supplementary Data, and they demonstrate that the model predictions were not sensitive to the assumptions made concerning respiration during the frozen period.

CO_2_ is driven out from the conduits by diffusion and further from inter-conduit air spaces to ambient air by diffusion, and also possibly during the freezing process by pressure-driven mass flow. The diffusion rate of a given gas is proportional to the concentration difference of that gas, and pressure-driven mass flow is proportional to the concentration difference of the gas multiplied by the pressure difference (Nobel, 2005). Pressure-driven mass flow from the inter-conduit spaces to the ambient air will rise if gas diffusion from the conduits to the inter-conduit air phase raises the gas pressure within the stem above atmospheric. The pressure increase during freezing propagation has been experimentally quantified by Robson and Petty (1987). In particluar, xylem and cambium present resistance to gas movement from the stem to ambient air (e.g. Teskey and McGuire, 2002), but do not completely prevent gas exchange (Sorz and Hietz, 2006; Steppe *et al*., 2007).

### Field data

Field data were collected at the SMEAR II station (Hari and Kulmala, 2005) located in southern Finland (61°51′N, 24°17′E), where stem CO_2_ efflux from a mature Scots pine tree has been continuously measured since 2003 using automated flow-through gas exchange cuvettes (Kolari *et al*., 2009). The transparent cuvette (3·5 × 20 cm) with a 1-cm-thick opaque rubber seal was attached to the north side of the stem on top of the bark. CO_2_ efflux was determined from the CO_2_ concentration increase in the cuvette measured with an infrared gas analyser (URAS 4; Hartmann & Braun, Frankfurt am Main, Germany) in a time frame varying from 30 to 60 min. We analysed the data from 2006 to 2009, from a cuvette situated at varying heights within the living crown. The studied cuvette was located at a height of 12 m in 2006 and 2008 and at a height of 13·7 m in 2007 and 2009. During this time period, the pine grew in height from 16·2 to 17·4 m and the crown base rose from 10 to 11 m.

We analysed the CO_2_ efflux response to the freezing events by combining the CO_2_ efflux data with xylem temperature and ambient temperature data measured at a height of 15 m near the tree where the cuvette was located. CO_2_ efflux decreases practically to zero once extracellular freezing is completed. We searched specifically for peaks in the CO_2_ efflux that occurred during the freezing process. From the whole data set, we selected 24 cases where CO_2_ efflux increased considerably with decreasing temperature (note that the change usually occurs in the same direction) after the temperature had dropped below zero. The magnitude of each freezing-related CO_2_ burst was calculated as the difference between measured CO_2_ efflux and modelled CO_2_ release, assuming that respiration continues its linear decrease with decreasing temperature from the level at the onset of the freezing-related CO_2_ burst to the post-burst level. Freezing-related CO_2_ burst duration was estimated visually from the figures.

## RESULTS

### Freezing-related CO_2_ bursts

Freezing-related CO_2_ bursts were clearly detected upon freezing in the laboratory experiments (Figs 1 and 2). The CO_2_ burst followed similar dynamics in each of the three repetitions in both studied conifers. It began 5 ± 1 min (mean ± s.d.) after the start of the freezing exotherm and continued for 37 ± 5 min.

The size of the freezing-related CO_2_ burst (in Fig. 1), i.e. the integral of the difference between measured CO_2_ efflux and the modelled CO_2_ release varied in absolute values from 177 to 1003 μmol m^–2^ (Table 1). The fraction of freezing-related CO_2_ burst from the stem ranged between 27 and 96 % of the total CO_2_ content within the stem (Table 1). On average 71 % of the CO_2_ within the stem before the onset of freezing was predicted to be burst out of the stem during freezing.

**Table 1.** Results for the freezing experiments presented for each repetition: diameter of the stem within the cuvette, stem CO_2_ content within the cuvette just prior to the freezing event, absolute size of the freezing-related CO_2_ burst connected to freezing and percentage of the CO_2_ burst compared with the total stem CO_2_ content

No.	Species	Diameter (cm)	Stem CO_2_ content before freezing (vol.%)	CO_2_ burst (μmol m^–2^)	Percentage of burst
1	*Pinus sylvestris*	0·75	0·4	549	83
2	*Pinus sylvestris*	0·76	0·6	890	84
3	*Pinus sylvestris*	0·90	0·6	469	44
4	*Picea abies*	0·85	0·5	1003	94
5	*Picea abies*	0·95	0·3	177	27
6	*Picea abies*	0·70	0·2	392	96

### Field measurements

**Fig. 3.**
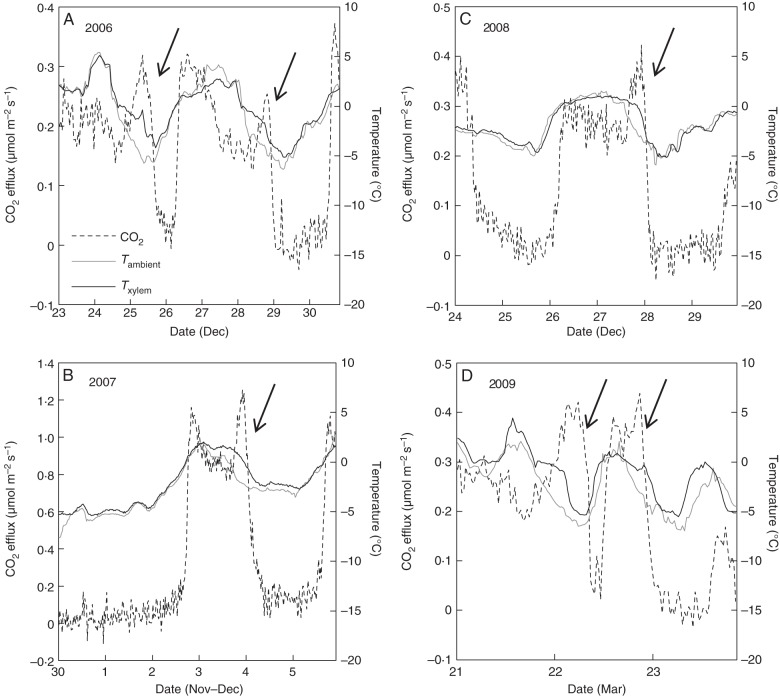
Time series of CO_2_ efflux measured from a Scots pine stem in the field during 2006–2009. Freezing-related CO_2_ bursts are marked with arrows. Ambient temperature was measured near the tree top, and xylem temperature from one location within the stem. Gas exchange cuvettes were located at heights of 12 m in A and C, and 13·7 m in B and D.

**Fig. 4.**
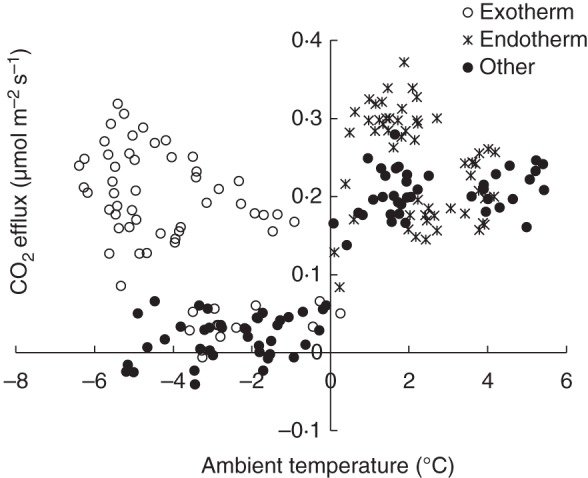
CO_2_ efflux measured from a Scots pine in the field in 2006, plotted against ambient temperature during freezing exotherms, thawing endotherms and other times. The data are the same as presented in Fig. 3A.

CO_2_ bursts induced by stem freezing were clearly also detected in the field measurements (Fig. 3). The size of the freezing-related CO_2_ burst averaged 5276 μmol m^–2^ (Table 2). The duration of the freezing-related CO_2_ burst was 9 ± 4 h. Clear CO_2_ bursts related to thawing were also visible in most cases (Fig. 3). Stem CO_2_ efflux was very close to zero with sub-zero temperatures, except during the freezing exotherms that correspond to the CO_2_ burst during freezing (Fig. 4). Stem CO_2_ efflux deviation during thawing can also be clearly distinguished, as the CO_2_ efflux is then considerably higher than during other times for a given temperature (Fig. 4).

**Table 2.** Field measurement results presented for each year (*n* cases selected per year): stem diameter at the location of the cuvette (different cuvette height each year), and mean size (±s.d.) of the CO_2_ burst connected to freezing

Year	*n*	Diameter (cm)	Mean CO_2_ burst (μmol m^–2^)
2006	5	7·3	3656 ± 2266
2007	4	5·9	10 246 ± 2045
2008	5	8·2	2956 ± 1402
2009	10	6·5	5258 ± 4382

## DISCUSSION

### Freezing-related CO_2_ burst

Clear increases in stem CO_2_ efflux were evident during freezing propagation in the xylem. The increased CO_2_ efflux was clearly detectable both in the saplings under laboratory conditions and in a mature pine in field conditions. We used a modelling analysis of the laboratory data to demonstrate that the freezing-related CO_2_ bursts were quite large (approx. 70 %) compared with the amount of dissolved gases within the stem. All gases are not trapped inside the ice within the stem as previously assumed. This is a new observation concerning the factors affecting winter embolism formation.

Stem CO_2_ efflux during the freezing processes has not been reported before as far as we know, but it has been reported that stem CO_2_ concentration is affected by unknown drivers under freezing conditions (Etzold *et al*., 2013). Etzold *et al.* (2013) found that fluctuations in stem CO_2_ concentration in Norway spruce could be explained with 80 % certainty by stem temperature as long as the stem was not frozen. But once the mean daily stem temperature decreased below 0 °C, CO_2_ concentration did not decrease with decreasing temperature as expected, but seemed to increase until the mean daily temperature decreased to approx. –2 °C (Etzold *et al*., 2013). The authors concluded that physically induced CO_2_ concentration changes could explain at least part of these results. This conclusion is supported by our findings and the theory (e.g. Sevanto *et al*., 2012) that freezing propagation concentrates gases within the stem. Freezing-related gas concentration has been proven experimentally via increased xylem pressure measurements (Robson and Petty, 1987). We also have consistently evidenced increased xylem pressures as temporal xylem swelling after freezing propagation has begun (L. Lindfors *et al*., unpubl. res.).

The size and temporal length differences of the freezing-related CO_2_ burst between laboratory and field measurements were of a similar magnitude considering the difference in stem size. Larger stems have larger volume (and more CO_2_) within the xylem per unit of stem surface area, and the length of the freezing process is determined mainly by the rate at which heat diffuses out of the stem. Both factors are dependent on the stem surface area to stem volume ratio, which in turn is inversely proportional to the stem radius (Ashworth *et al*., 1985; Pramsohler *et al*., 2012). Considering that the stem diameter was approx. 10 times larger in our field measurement in relation to the laboratory measurements, the 10 times larger and 15 times longer freezing-related CO_2_ burst in the field compared with the saplings used in the laboratory measurements seems logical. Variation in the CO_2_ burst size was surprisingly large between individuals measured in the laboratory and between freezing events measured in the field, which we cannot explain.

We used the model to estimate that the CO_2_ concentration in small saplings was 0·2–0·6 % of the gaseous stem volume prior to the beginning of freezing. As expected, these values are lower than results reported for mature Scots pine (3–20 %; Hari *et al*., 1991) and Norway spruce (2–10 %; Eklund, 1990) during the active period. CO_2_ concentration measured in the gaseous volume for mature Norway spruce during dormancy was also higher (approx. 4 %; Etzold *et al*., 2013) compared with our results. However, larger stems have a longer CO_2_ diffusion distance out of the stem to ambient air. Thus, larger CO_2_ concentration differences between the stem and ambient air are required for the respired CO_2_ to diffuse out of the stem, provided that area-specific respiration does not change with tree size. It is also known that CO_2_ concentrations within the stem are not spatially homogeneous but form a radial gradient within the stem, with higher concentrations found in the inner parts of the stem compared with the outermost xylem (Chase, 1934). In fact, CO_2_ would not diffuse out of the stem at all without this radial concentration gradient. This makes the estimation of total stem CO_2_ content difficult based on spatially limited empirical measurements (Hölttä and Kolari, 2009).

We modelled the stem respiration rate during freezing propagation by linearly extrapolating respiration during freezing propagation down to the level measured after the CO_2_ efflux had settled to a constant level in a frozen stem. It is not known how respiration responds to temperatures below the freezing point. We therefore tested two additional scenarios to model respiration, shown in detail as Supplementary Data: (A) freezing had no effect on the temperature dependency of the respiration rate, and (B) respiration was dropped to zero after the start of the freezing exotherm. The average fraction of the freezing-related CO_2_ burst was 59 and 88 %, respectively, in scenarios A and B (Supplementary Data).

In general, maintenance of respiration is known to depend on temperature through enzymatic degradation processes (Thornley and Johnson, 1990). However, earlier studies have shown that stem respiration also drops with decreasing stem water potential (Wang *et al*., 2003; Saveyn *et al*., 2007, 2008). It is well known that water potential over ice decreases 1·2 MPa per degree decrease in ice temperature (Washburn and West, 1928; Rajashekar and Burke, 1982). Thus, the temperatures experienced by the living cells in our laboratory experiment are causing extremely high water stress, equivalent to a sharp drop in water potential down to –5 MPa at freezing onset. Water potential over ice decreased further to –12 MPa with decreasing temperature. Thus, it is very possible that the respiration rate would in reality decrease even more sharply than modelled in this paper, in which case the freezing-related CO_2_ burst would represent an even greater share of the measured CO_2_ efflux.

During the last decade it has been acknowledged that a large proportion of respired CO_2_ is not actually released as CO_2_ efflux at the production site. Instead, a large proportion is transported with the xylem sap (Teskey and McGuire, 2007; Hölttä and Kolari, 2009; Bloemen *et al*., 2013). In our study, the transport of CO_2_ with the xylem sap should be negligible due to low sap flow rates at the low temperatures experienced, and also due to lack of light in the laboratory experiment, both factors decreasing the transpiration rate (see Teskey and McGuire, 2007; Bloemen *et al*., 2013).

It can be seen from both the laboratory and the field data that stem CO_2_ efflux decreases practically to zero when the stem is frozen. CO_2_ efflux from the frozen stem may be zero for two reasons: (1) respiration is absent, or (2) the respired CO_2_ is unable to diffuse out from the stem while the xylem conduits are frozen. If the latter was the case, then we would expect to see large CO_2_ bursts from the stem during thawing as CO_2_ would have accumulated in the stem during the frozen period. CO_2_ bursts unexplained by temperature were indeed detected also during thawing. However, it is also likely that some fraction of the freezing-related CO_2_ burst is trapped in intercellular spaces in the frozen stem together with respired CO_2_ and contributes to the bursts measured during thawing. Our results also indicate that respiration continues rapidly after thawing.

We measured stem CO_2_ efflux in a mature Scots pine in the field with transparent cuvettes, which means that non-foliar photosynthesis can affect the net CO_2_ efflux (Pfanz and Aschan, 2001). Thus, we cannot rule out the possibility of re-fixation of some of the respired CO_2_ during the freezing process, but that does not weaken our results relating to CO_2_ burst during freezing. In practice, stem surface photosynthesis was probably nearly absent, as only a small fraction of photosynthetically active radiation reaches the cuvette (attached to the north side of the stem) through the canopy and further the stem surface inside the narrow cuvette, and given that stem surface photosynthesis is more evident in twigs and young branches than in stem surrounded by a thicker layer of dead bark (Pfanz and Aschan, 2001).

Xylem sap also contains other dissolved gases in addition to CO_2_, namely N_2_ and O_2_. All of these gases can be expected to contribute to winter embolism formation. We were able to quantify only the amount of CO_2_ efflux from the stem during freezing propagation. However, the phenomenon should be qualitatively equivalent for the other gases, taking into account that their solubility, diffusion coefficient and concentrations in the xylem sap and ambient air differ from each other.

### Bubble formation during the freezing process

An empirically shown link exists between conduit size and the degree of winter embolism (Sperry and Sullivan, 1992; Sperry *et al*., 1994; Davis *et al*., 1999; Pittermann and Sperry, 2003; Wheeler *et al*., 2005; Wilson and Jackson, 2006), and this link has been explained by the equivalence between conduit volume and the volume of gases within the conduit. However, Sperry and Sullivan (1992) have shown that the critical tensions causing embolism during thawing calculated for a given conduit size are an order of magnitude lower than those actually measured. Our hypothesis that the difference between theoretical and measured critical tensions can be explained by the efflux of gases from the conduits was confirmed by our results. Pittermann and Sperry (2006) also reported that decreasing conduit size gave more safety against winter embolism than could be explained by the decreased water volume alone. Our results imply that conduit water volume alone does not determine the volume of bubbles forming during freezing, but also the degree of freezing-related gas efflux from the conduit.

The mechanism of bubble formation during freezing propagation could be proposed as follows: the moving ice front concentrates the dissolved gases in front of itself, as a given amount of gases must be constrained to a smaller volume (Sevanto *et al*., 2012). The increased concentration of the dissolved gases leads to ‘competition’ between two processes: diffusion of the dissolved gases out from the conduit to air spaces inside the stem and bubble nucleation. In the case of small conduit size, gas efflux is accelerated to the interconduit spaces due to the large conduit surface area relative to conduit volume, i.e. the dissolved gas is on average closer to the conduit surface in the small conduit. Gases are further extracted from the interconduit spaces out from the stem by diffusion and pressure-driven mass flow. This process is dependent on stem surface area relative to its volume. Our results show that, although the cell wall and bark slow down diffusion, diffusion is not blocked (Sorz and Hietz, 2006) contrary to the theory of Pittermann and Sperry (2006) that assumes all gases to be trapped inside the conduits during freezing.

In the case of large conduits, the probability of bubble nucleation during freezing is likely to be higher in comparison with the small conduits. The probability of bubble nucleation increases when the concentration of the dissolved gases increase and more time is given for the nucleation to occur (Brennen, 1995). First, the concentration of gases can be expected to be higher in large than in small conduits due to lower diffusion efficiency. Diffusion rate is inversely proportional to diffusion distance, and the characteristic diffusion time is inversely proportional to the square of distance (e.g. Nobel, 2005). Bubble nucleation will occur if the concentration of dissolved gases increases above a certain threshold value. In practice, the probability of bubble nucleation will rise sharply and highly non-linearly when super-saturations exceed a few tens of atmospheres (Sevanto *et al*., 2012). Secondly, more time is given for bubble nucleation to occur in larger conduits compared with smaller ones due to the lower ice propagation rate. There is a clear physically based relationship between ice propagation rate and ice nucleation temperature (Kitaura, 1967; Hacker and Neuner, 2007; Hacker *et al*., 2011) and a recent study has shown that ice nucleation temperature is higher in large conduits than in small ones (Lintunen *et al*., 2013).

To conclude, we have shown that a large proportion of gas dissolved in the xylem sap is released from the stem during freezing. It has previously been assumed that all gas dissolved in the xylem sap is trapped within the stem and forms bubbles. Extraction of gases during freezing can be beneficial for a plant in avoiding winter embolism. Gas extraction during freezing can be hypothesized to increase the critical conduit diameter that causes winter embolism.

## SUPPLEMENTARY DATA

Supplementary data are available online at www.aob.oxfordjournals.org and consist of model results with two alternative respiration scenarios.

Supplementary Data
