# Supplementary material for: Bursts of CO2 released during freezing offer a new perspective on avoidance of winter embolism in trees
Source: Ann Bot. 2014 Sep 24;114(8):1711–8. doi: 10.1093/aob/mcu190 (PMC4649691; doi:10.1093/aob/mcu190)
Supplement: Supplementary Data [file supp_114_8_1711__index.html]

Bursts of CO2 released during freezing offer a new perspective on avoidance of winter embolism in trees — Supplementary Data 

# Bursts of CO2 released during freezing offer a new perspective on avoidance of winter embolism in trees

## Supplementary Data

Supplementary Data

**Files in this Data Supplement:**

- Supplementary Data - Pdf file
