# Supplementary material for: Bursts of CO2 released during freezing offer a new perspective on avoidance of winter embolism in trees
Source: Ann Bot. 2014 Sep 24;114(8):1711–8. doi: 10.1093/aob/mcu190 (PMC4649691; doi:10.1093/aob/mcu190)
Supplement: Supplementary Data [file supp_mcu190_mcu190supp.pdf]

SUPPLEMENTARY DATA

Alternative respiration scenarios during freezing

We assumed in the manuscript that respiration decreased linearly during the freezing propagation down to the level measured after the CO_2 efflux had settled to a constant level in a frozen stem. However, there is very little information on the temperature dependency of respiration in a frozen stem. Therefore we represent here two alternative scenarios: (A) freezing had no effect on the temperature-dependency of respiration rate; and (B) respiration was dropped to zero after the start of the freezing (Fig. S1).

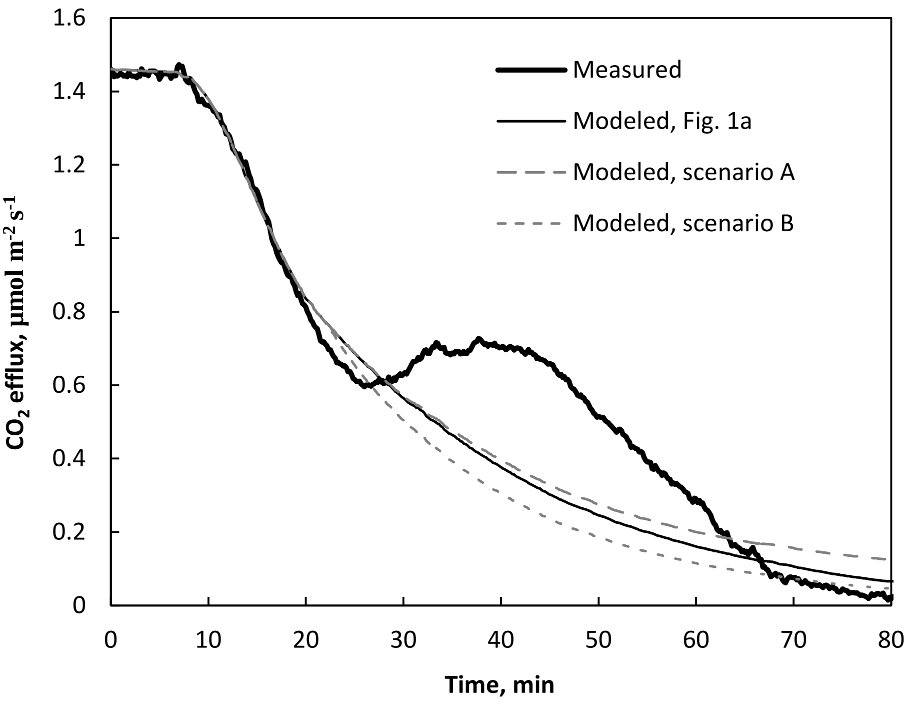

Figure S1. Measured CO_2 efflux during the freezing experiment is shown for tree no. 3 together with three model estimates for stem CO_2 release if respiration would be the only source of CO_2. One of the model estimates is as presented in Fig. 1A. The two other estimates are based on different scenarios regarding behavior of respiration input during freezing propagation. In scenario (A), freezing had no effect on the temperature-dependency of respiration rate, and in scenario (B), respiration was dropped to zero after the start of the freezing exotherm. The difference between the measured CO_2 efflux and modeled CO_2 release represents the freezing-related CO_2 burst out of the stem.

The fraction of freezing-related burst of CO_2 from the stem was between 18 and 76% and between 37 and 119% of the CO_2 content inside the stem, in scenarios (A) and (B), respectively (Table S1). Depending on the scenario, on average 59 or 88% of the CO_2 inside the stem before the onset of freezing was predicted to be pushed out from the stem during freezing.

Table S1. Results for the freezing experiments presented for each repetition: absolute size of the freezing-related burst of CO_2 connected to freezing in alternative scenarios (A) and (B) and percentage of the CO_2 burst compared to the CO_2 content of the stem in alternative scenarios (A) and (B).

Tree no.	Species	CO_2 burst (µmol m^{-2})		Percentage of burst, %	
		A	B	A	B
1	*P. sylvestris*	480	647	72	97
2	*P. sylvestris*	775	1048	73	99
3	*P. sylvestris*	422	619	39	58
4	*P. abies*	811	1274	76	119
5	*P. abies*	114	241	18	37
6	*P. abies*	303	488	74	119
